# Microbiomes in physiology: insights into 21st‐century global medical challenges

**DOI:** 10.1113/EP090226

**Published:** 2022-02-14

**Authors:** Emad Shehata, Aimée Parker, Toru Suzuki, Jonathan. R. Swann, Jotham Suez, Paul. A. Kroon, Priscilla Day‐Walsh

**Affiliations:** ^1^ Quadram Institute Bioscience Food Innovation and Health & Gut Microbes in Health and Disease Programmes Quadram Institute Bioscience Norwich UK; ^2^ Chemistry of Flavour and Aroma Department National Research Centre Dokki Cairo Egypt; ^3^ Department of Cardiovascular Sciences and NIHR Leicester Biomedical Research Centre University of Leicester Glenfield Hospital Leicester UK; ^4^ Faculty of Medicine School of Human Development and Health University of Southampton Southampton UK; ^5^ W. Harry Feinstone Department of Molecular Microbiology and Immunology Johns Hopkins Bloomberg School of Public Health Baltimore MD USA

**Keywords:** antibiotic resistance, cardiovascular physiology, faecal microbiota transplantation, inflammaging, irritable bowel syndrome, neuroimmunology, probiotics, resistome

## Abstract

**New Findings:**

- **What is the topic of this review?**
  The role of the gut microbiome in physiology and how it can be targeted as an effective strategy against two of the most important global medical challenges of our time, namely, metabolic diseases and antibacterial resistance.
- **What advances does it highlight?**
  The critical roles of the microbiome in regulating host physiology and how microbiome analysis is useful for disease stratification to enable informed clinical decisions and develop interventions such as faecal microbiota transplantation, prebiotics and probiotics. Also, the limitations of microbiome modulation, including the potential for probiotics to enhance antimicrobial resistance gene reservoirs, and that currently a ‘healthy microbiome’ that can be used as a biobank for transplantation is yet to be defined.

**Abstract:**

The human gut microbiome is a key factor in the development of metabolic diseases and antimicrobial resistance, which are among the greatest global medical challenges of the 21st century. A recent symposium aimed to highlight state‐of‐the‐art evidence for the role of the gut microbiome in physiology, from childhood to adulthood, and the impact this has on global disease outcomes, ageing and antimicrobial resistance. Although the gut microbiome is established early in life, over time the microbiome and its components including metabolites can become perturbed due to changes such as dietary habits, use of antibiotics and age. As gut microbial metabolites, including short‐chain fatty acids, secondary bile acids and trimethylamine‐*N*‐oxide, can interact with host receptors including G protein‐coupled receptors and can alter host metabolic fluxes, they can significantly affect physiological homoeostasis leading to metabolic diseases. These metabolites can be used to stratify disease phenotypes such as irritable bowel syndrome and adverse events after heart failure and allow informed decisions on clinical management and treatment. While strategies such as use of probiotics, prebiotics and faecal microbiota transplantation have been proposed as interventions to treat and prevent metabolic diseases and antimicrobial resistance, caution must be exercised, first due to the potential of probiotics to enhance antimicrobial resistance gene reservoirs, and second, a ‘healthy gut microbiome’ that can be used as a biobank for transplantation is yet to be defined. We highlight that sampling other parts of the gastrointestinal tract may produce more representative data than the faecal microbiome alone.

## 1 INTRODUCTION

The microbiome is critical to physiological homoeostasis, influencing health and disease status in the host. The human body contains trillions of microbes encompassing bacteria, archaea, viruses and microeukaryotes (Zhang et al., 2019). In the current report, the gut microbiome consists of symbiotic or pathobionts that are resident in the host and opportunistic pathogens that are acquired from the environment or other parts of the body (Casadevall & Pirofski, 1999; Chow et al., 2011). While opportunistic pathogens can cause acute effects, pathobionts are only able to cause deleterious effects to host health in certain circumstances, such as when the immune system has become compromised. Commensals/symbionts contribute to the maintenance of physiological homoeostasis as well as providing colonisation resistance to opportunistic pathogens (Hornef, 2015). Acute deleterious effects of pathogens in physiology can lead to infections and in extreme cases cause bacteraemia leading to sepsis and death. This has led to heavy overuse of antibiotics to combat infections, which has provided the selective pressure that is driving increases in antibiotic resistance (Ahmed, 2005; Casadevall & Pirofski, 1999; Lau et al., 2004).

As well as harbouring antimicrobial resistance genes, the gut microbiome may also influence systemic physiological functions by competing for essential nutrients or digesting complex molecules to produce substrates for host energy metabolism and cell signalling (Martin et al., 2019). The gut microbiota can therefore also cause subtle but chronic physiological effects, which contribute to the epidemic of metabolic/inflammatory diseases such as diabetes, cardiovascular disease and neurodegenerative diseases (Suez et al., 2018; Wang et al., 2020). Coupled to antibiotic resistance, metabolic diseases are among the leading global medical challenges of our time, posing a socio‐economic burden worldwide. Nevertheless, mechanisms through which the microbiome influences physiology remain relatively poorly understood. It is envisaged that advances in sampling techniques, multi‐omic approaches (genomic, transcriptomic, methylomic, proteomic, metabolomic), and bioinformatic tools will increase the resolution at which these pan‐kingdom interactions can be studied, thus expanding our understanding of the influence of the microbiome on host physiology in health and disease. Such advances will likely revolutionise future clinical practices in disease prevention, treatment and management.

The most compelling evidence for the influence of microbes on human physiology comes from bacterial/viral infections, where a coordinated systemic reaction that evokes a signalling cascade is manifested by a raised body temperature, muscle weakness and pain, and, if poorly managed, results in multi‐organ failure and subsequently death (Stearns‐Kurosawa et al., 2011). Thus, understanding systemic host responses not only to exogenous microbes but also the microbiome in general is critical. Whether microbiomes are favourable to the host depends on the types and strains that make up the microbiome species and how they interact with the host and other members of the microbial community, which is further influenced by factors such as diet, general health and the environment. The focus of the “Microbiomes in Physiology” symposium, which took place virtually on the 14th July 2021 at the main Physiological Society meeting and the current report is on the role of the gut microbiome in regulating physiological functions locally in the gut and remotely in various gut–organ axes, including the heart, the liver and the brain.

## 2 MICROBES ACROSS THE LIFESPAN

The overall composition of the microbiome is determined by early life events such as mode of delivery, breastfeeding and frequency of antibiotic use. However, the abundance of each microbe may fluctuate across the lifespan due to factors such as age, diet, lifestyle, cultural practices and geographical location (Arboleya et al., 2012; Johnson & Versalovic, 2012). In general, greater microbial diversity and functional redundancy provide resilience to perturbation by the aforementioned factors, and therefore are associated with beneficial impacts on the health of the host (Vieira‐Silva et al., 2016).

The presence of microbial genes within the host critically impacts on host metabolic fluxes, with the production of certain metabolites being detrimental and of others beneficial to the health of the host. Microbial metabolism of certain essential dietary precursors can also confer a competitive nutrition partitioning environment between the host and the microbiome. Both microbiota composition and the resulting biochemical products have been shown to change over the life course. For example, the metabolic capacity of the intestinal microbiota to degrade complex carbohydrates to short‐chain fatty acids (SCFAs) declines with age, while the capacity to transform essential nutrients and proteins into toxic compounds such as trimethylamine‐*N*‐oxide (TMAO) and indole sulphates increases with age (Agus et al., 2021; Lee et al., 2020; Rios‐Covian et al., 2020). At the symposium, Swann discussed age‐dependent variability in the neurobiochemical profiles of mice across the lifecourse, with fluctuations in several microbially derived metabolites. This included metabolites such as 3‐indoxyl‐sulphate, γ‐aminobutyric acid, TMAO, hippurate and phenylacetylglutamine (Swann et al., 2020). While certain metabolites were abundant during the neonatal period and declined into adulthood, others gradually increased with age, and some peaked in abundance at puberty before returning to neonatal levels in adulthood (Swann et al., 2020). As many of these compounds are involved in brain function and development, it is important to characterise whether these fluctuations and their timings would affect developmental plasticity, neonatal growth trajectory and the risk of disease in both childhood and adulthood. Indeed, exposure to certain environmental and nutritional cues during critical periods of growth and development have been shown to influence the risk of developing disease both in early life and in adulthood according to the ‘thrifty phenotype’ or ‘the developmental origins of health and disease’ hypothesis (Farshim et al., 2016; Hales & Barker, 2001; Hanson & Gluckman, 2014; Osman et al., 2021).

**SYMPOSIUM HIGHLIGHTS**

1. The core microbial composition and diversity of the gut is established early in life (within 3 years after birth). Microbial diversity, composition and function can fluctuate over the life course with alterations in microbial metabolite production during critical periods of development contributing to chronic diseases in both childhood and adulthood.
2. While to date a ‘healthy microbiome’ has not been defined owing to intra‐ and inter‐individual variations in the core microbiome, various microbial products can be used, due to their common functionality, to stratify host disease phenotypes such as irritable bowel syndrome and cardiovascular disease and can predict clinical outcomes after hospitalisation from diseases such as heart failure.
3. Plasma trimethylamine *N*‐oxide (TMAO) is an example of a microbially derived metabolite shown to be an important prognostic marker of adverse events after heart failure and all‐cause mortality and is comparable to traditional markers such as B‐type natriuretic peptide (BNP) and N‐terminal (NT)‐pro‐hormone BNP (NT‐proBNP). Currently, there are no specific drugs that effectively and specifically alter the microbiome to reduce TMAO burden.
4. While there is a debate as to whether alterations in microbial structure and their metabolites such as TMAO may be causative, a mere correlation or indeed a symptom of disease, recent studies using faecal microbiota transplantation and conventionalised animal models indicate that the microbiome has direct/causative effects on host physiology, including effects on inflammaging. The supplementation of TMAO has direct atherogenic effects. In general, high microbial trimethylamine (TMA) production is indicative of altered microbiome composition and structure.
5. The microbiome can also affect the host's health by supporting pathogenic bacteria. This may be mediated by metabolic cross‐feeding, whereby commensal species produce metabolites, which support the growth of pathogens and pathobionts. In addition, the microbiome can serve as a reservoir for antimicrobial resistance genes, which can transfer horizontally to pathogenic bacteria. While probiotics have been widely used to prevent or treat diseases (potentially through the modulation of the microbiome), and have been postulated to reduce resistance genes, it has been shown that probiotics may increase and exacerbate the number of antimicrobial resistance genes enhanced by antibiotics.
6. Most data on the diversity and function of the microbiome have been inferred from sampling the faecal microbiome but emerging evidence suggests that the faecal microbiome may misrepresent effects of probiotics on the intestinal microbiome community and the abundance of resistance genes in the gastrointestinal (GI) tract. Therefore, the use of direct sampling from the GI tract is paramount in future research.

At the symposium, a direct impact of the microbiome on age‐associated inflammation in the brain was described by Parker who reported that faecal microbiota transplantation (FMT) treatment was effective in switching from an age‐associated chronic low‐grade inflammatory phenotype (inflammaging) and a younger less inflammatory phenotype (Parker et al., 2021). Young mice receiving microbiota from aged mice exhibited an elevated inflammatory phenotype, whereas age‐associated serum and brain inflammatory changes in mice could be reduced or reversed by transplantation with microbiota from young donor mice (Parker et al., 2021). Regulatory effects of the FMT treatments were observed in the intestinal epithelial barrier and in the retina. The authors identified serotonergic signalling together with altered lipid and vitamin metabolism as possible mechanisms through which the microbiome may influence age‐associated inflammation and functional decline in the gut and the central nervous system. The debate over whether microbes directly impact on physiology has been compounded by limited knowledge available on the mechanisms through which microbially derived compounds alter physiological homoeostasis. For example, while in some cases, TMAO produced by the microbiome has been suggested to play an important role in neural development, others have demonstrated an influence of TMAO on brain ageing and cognitive decline (Li et al., 2018; Vuong et al., 2020). Additionally, there are a substantial number of studies showing associations between high plasma levels of TMAO and metabolic diseases, as well as adverse secondary events after heart failure with further studies showing direct atherogenic effects of TMAO in both humans and mice (Brunt et al., 2020; Geng et al., 2018; Tan et al., 2019).

## 3 MICROBIAL COMPONENTS, CELL SIGNALLING AND DISEASE STRATIFICATION

**FIGURE 1:**
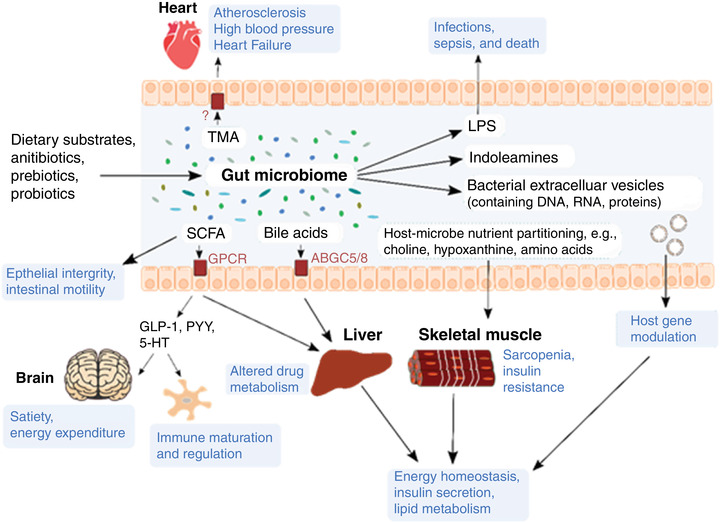
Effects of microbially derived components on host physiology. GLP‐1, glucagon‐like peptide‐1; LPS, lipopolysaccahride; PYY, peptide YY

In their talks, Swann and Parker highlighted various signalling pathways that are affected by microbially derived compounds (such as metabolites, cell wall components and extracellular vesicles), which can regulate immune function, metabolic homoeostasis and brain function (Figure 1). Among the metabolites, SCFAs are perhaps the most‐studied gut microbially derived metabolites. SCFAs interact with a range of receptors such as G protein‐coupled receptors (GPCRs) on host cells, both locally in the gut and in remote organs such as the brain, heart and the liver. Through their interaction with GPCRs, SCFAs have been shown to modulate the secretion of hormones including glucagon‐like peptide‐1 and peptide YY, which impact on the brain functions such as mood, appetite, food intake and energy expenditure (Frost et al., 2014; Modasia et al., 2020). In the gut, SCFAs are also utilised by intestinal epithelial and colonic cells as energy sources, positively promoting gut barrier integrity, as well as maintaining low intestinal pH that is unfavourable to opportunistic pathogens and pathobionts (Pérez‐Reytor et al., 2021). Nevertheless, SCFAs may also be used by pathogens such as *Salmonella*, *Clostridium* and *Citrobacter* species as a cue for expressing virulence genes (Zhang et al., 2020). This is a particularly good example of how the interaction of specific microbial species with the complex multi‐organism gut microbiome may influence disease risk. In irritable bowel syndrome (IBS) patients with constipation (IBS‐C), reduced levels of SCFAs in faecal samples are coupled with reduced levels of acetate in mucosal biopsies (Mars et al., 2020). Although SCFAs derive from dietary fibre, these observations were independent of dietary fibre intake suggesting that other factors may influence the availability of SCFAs. Recent data suggest that SCFAs may derive from the metabolism of l‐carnitine to trimethylamine, a pathway highlighted by Suzuki at the symposium (Suzuki et al., 2021) and discussed below in relation to the atherogenic phenotype (Rajakovich et al., 2021). In their talk, Swann further demonstrated that in contrast to IBS‐C patients, IBS patients with diarrhoea (IBS‐D) present higher levels of tryptophan and its indoleamine microbial metabolite, tryptamine, which again acts locally to regulate intestinal motility by interacting with serotonin receptor‐4 (Swann et al., 2020). This was coupled with increased amounts of unconjugated bile acids and decreased amounts of primary bile acids in IBS‐D patients (Mars et al., 2020). Another microbial metabolite, hypoxanthine, provides an excellent example of competitive nutritional partitioning between the microbiota and the host (Swann et al., 2020). Hypoxanthine is an important energy source for intestinal epithelial cells, promoting epithelial cell development and recovery from injury, however, Swann demonstrated increased hypoxanthine use by the gut microbiome with decreased levels being observed in IBS‐C patients (Mars et al., 2020). As such, alterations in microbiota composition and metabolites during critical developmental periods may prove detrimental to health.

## 4 STRATIFYING DISEASE OUTCOMES AND CLINICAL DECISIONS BASED ON MICROBIAL METABOLITES: THE CASE OF TRIMETHYLAMINE OXIDE AND HEART FAILURE

**FIGURE 2:**
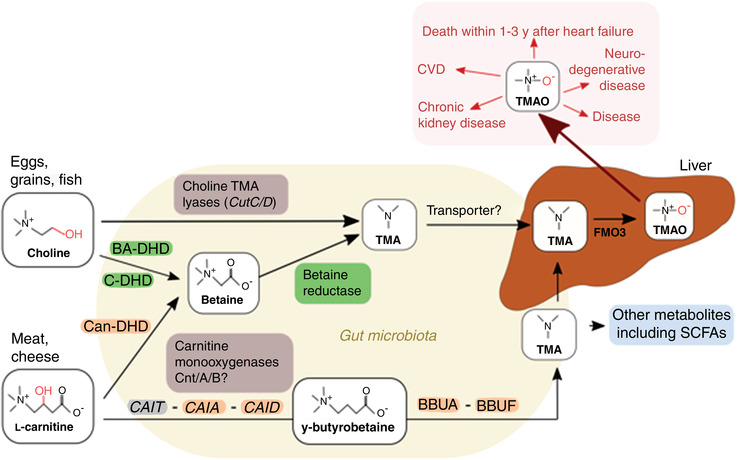
Mechanisms of TMA production by the gut microbiota. BA‐DH, betaine aldehyde dehydrogenase; BBUA‐BBUF, gamma‐butyrobetaine utilization genes A to F; C‐DH, CAIA, crotonobetainyl‐CoA reductase; CAID, carnitinyl‐CoA dehydratase; CAIT, carnitine/gamma‐butyrobetaine antiporter; Can‐DH, carnitine dehydrogenase choline dehydrogenase; Cnt/A/B, carnitine monooxygenase oxygenase subunits A/B; CutC/D, choline trimethylamine‐lyase C/D; CVD, cardiovascular disease; FMO3, flavin monooxygenase 3

The possibility of stratifying patients and guiding clinical decisions based on metabolic profiles was highlighted by Suzuki. Plasma TMAO levels were shown to be a strong predictor of adverse secondary events after heart failure compared to traditional markers such as N‐terminal pro‐B‐type natriuretic peptide (NT‐proBNP) (Senthong et al., 2016; Suzuki et al., 2016). TMAO is produced in the liver by flavin‐containing monooxygenase isoform 3 (FMO3) following oxidation of trimethylamine (TMA), a derivative of essential dietary components l‐carnitine and choline, found in high quantities in red meat and eggs, respectively (Figure 2) (Koeth et al., 2013). The metabolism of choline to TMA seems to involve the direct choline‐TMA lyase pathway (Day‐Walsh et al., 2021). However, the metabolism of l‐carnitine involves the formation of an obligate intermediate, γ‐butyrobetaine, which is further metabolised in a multistep process involving several gene clusters to produce TMA and other metabolites including SCFAs, such as acetate and butyrate, which have been shown to be the end‐products in this process (Day‐Walsh et al., 2021; Rajakovich et al., 2021). The factors that regulate the formation of TMA from carnitine are yet to be understood although it seems that this pathway may be more important in the production of the atherogenic TMA than that involving choline. In their talk, Suzuki further highlighted that the associations of TMAO with adverse events after heart failure are influenced by geographical location, being higher in individuals from Norway, the Netherlands, Germany, Sweden and the United Kingdom than in those from Italy and Greece (Suzuki et al., 2019). Of note, this geographical variation was shown to be independent of polymorphisms in the *FMO3* gene along with diet, indicating that there is a yet unknown factor influencing the predictive capacity of TMAO on all‐cause mortality and death after heart failure.

To date, it has been difficult to clarify the compositions or indeed microbial species that can be used to predict disease phenotypes in the host. However, the abundant microbial metabolites provide an opportunity to profile and characterise individuals who may be at risk of not only heart failure but other metabolic diseases as well as hospitalisation and adverse events after hospitalisation including death. In their talk, Suzuki demonstrated that heart failure patients presenting high levels of TMAO when they are admitted to the hospital might still have high levels after treatment; that is to say, current treatments of heart failure patients and management of their future risk do not target their TMAO status. This presents an opportunity to stratify patients who may be at risk and to make clinically relevant informed decisions based on microbial metabolites. Nevertheless, there is an urgent need for therapies that can target the microbiome to reduce the burden of TMAO in those at risk, as current medications such as β‐blockers do not target the microbiome.

## 5 MANIPULATING THE MICROBIOME FOR THE BENEFIT OF THE HOST: PROBIOTICS, PREBIOTICS AND FAECAL MICROBIOTA TRANSPLANTATION

Supplementation with live probiotic microorganisms has been proposed as a means for beneficially altering the microbiome, for example by reducing the production of disease‐associated metabolites (including TMAO) or reducing the burden of pathobionts and commensals carrying antibiotic resistance genes. As highlighted by Swann probiotics, including those commonly used as dietary supplements, could support health by preventing the colonisation of pathobionts in preterm babies (Alcon‐Giner et al., 2020). However, in their talk, Suez highlighted the complexity and limitations of using probiotics in adults, as the colonisation success of supplemented probiotics shows high inter‐individual variations, which may underlie heterogeneity in probiotics’ efficacy (Zmora et al., 2018). For example, members of the gut microbiome encode for antibiotic resistance genes, creating a reservoir (resistome) that can transfer horizontally to pathogens and pathobionts, facilitating the emergence of antibiotic‐resistant strains. In their work, Suez demonstrated that probiotics can reduce the reservoir of antibiotic resistance genes in the human gut, but only in individuals permissive (receptive) to probiotic colonisation (Montassier et al., 2021).

In addition to being a major contributor to the expansion of the gut resistome, the use of antibiotics perturbs the gut microbiome, leading to dysbiosis associated with an elevated risk for non‐communicable diseases. Probiotics are often consumed in conjunction with antibiotic therapy to prevent detrimental effects of antibiotics on the microbiome. In their talk, Suez reported that, surprisingly, probiotics delay, rather than facilitate recovery of microbiome diversity from a course of antibiotics. Furthermore, probiotics contributed to an expansion in the number of antibiotic resistance genes in the gut, and in particular increased the abundance of the clinically relevant vancomycin resistance gene (*VanG*) (Suez et al., 2018).

In addition to probiotics, nutraceutical compounds such as complex carbohydrates and polyphenols have been investigated for their capacity to alter the microbiome for the benefit of the host (prebiotics). While the increase in certain microbes in response to these nutraceuticals may suggest beneficial effects, caution has to be exercised as metabolic cross‐feeding may promote the growth of a beneficial species, which will consequently produce metabolites that facilitate the survival of pathobionts (Eloe‐Fadrosh & Rasko, 2013; Mohajeri et al., 2018). FMT has also been proposed as a mode to increase microbial diversity or rebalance a dysbiotic microbial composition resulting from infection or ageing for example. As demonstrated by Parker et al., FMT from young donors proved effective in preventing age‐associated symptoms and inflammation (Parker et al., 2021). In the context of the resistome, Suez demonstrated that FMT was more effective than probiotic supplementation at restoring the resistome back to pre‐antibiotic status. However, the complexity of the microbiome and variations between individuals make it difficult to pinpoint a ‘healthy’ or optimal microbial composition that can be used as a biobank to treat all those with gut dysbiosis.

## 6 CURRENT LIMITATIONS AND THE FUTURE OF THE MICROBIOME IN MEDICAL PHYSIOLOGY

The microbiome offers a unique albeit challenging opportunity to improve host metabolic physiology and revolutionise future clinical practices in disease prevention, treatment and management. However, there was a consensus among the speakers at the symposium on the requirement for the standardisation of sampling and experimental approaches, which will greatly improve our ability to understand the role of the microbiome in physiology. In particular, Swann highlighted the need for averaging longitudinal data from an individual collected from multiple sampling points while Suez demonstrated the disparity between the microbiome and resistome within the stool sample and that from different sites within the gastrointestinal tract. To date, most research has focused on the bacterial component (bacteriome) of the microbiome, but there is an increasing appreciation of the importance of the viral (virome) and fungal (mycobiome) fractions of the microbiome, which is likely to become more apparent as our ability to study these elements evolves, in particular as the bacteriome along with its metabolome is also substantially influenced by phage predation (Hsu et al., 2019).

Thus, it is evident that many of the non‐communicable diseases proposed to be influenced by the microbiome coupled to antimicrobial resistance pose major socio‐economic challenges. Understanding the role of the microbiome in physiology and how it can be harnessed to underpin the development of effective therapies and preventative treatments will require a coordinated multidisciplinary research effort by physiologists, microbiologists, nutritionists, clinicians and partnerships with commercial organisations.

## COMPETING INTERESTS

Authors declare no conflict of interest.

## AUTHOR CONTRIBUTIONS

Conception or design of the work: P.D.‐W. and E.S. Acquisition, analysis or interpretation of data for the work: P.D.‐W., A.P., T.S., J.R.Sw., J.Su., P.A.K. and P.D.‐W. Drafting of the work or revising it critically for important intellectual content: P.D.‐W., A.P., T.S., J.R.Sw., J.Su., P.A.K. and P.D.‐W. All authors have read and approved the final version of this manuscript and agree to be accountable for all aspects of the work in ensuring that questions related to the accuracy or integrity of any part of the work are appropriately investigated and resolved. All persons designated as authors qualify for authorship, and all those who qualify for authorship are listed.

## References

[eph13146-bib-0001] Agus, A. , Clément, K. , & Sokol, H. (2021). Gut microbiota‐derived metabolites as central regulators in metabolic disorders. Gut, 70(6), 1174–1182. 10.1136/gutjnl-2020-323071 33272977 PMC8108286

[eph13146-bib-0002] Ahmed, N. (2005). 23 years of the discovery of *Helicobacter pylori*: Is the debate over? Annals of Clinical Microbiology and Antimicrobials, 4, 17. 10.1186/1476-0711-4-17 16262889 PMC1283743

[eph13146-bib-0003] Alcon‐Giner, C. , Dalby, M. J. , Caim, S. , Ketskemety, J. , Shaw, A. , Sim, K. , Lawson, M. A. E. , Kiu, R. , Leclaire, C. , Chalklen, L. , Kujawska, M. , Mitra, S. , Fardus‐Reid, F. , Belteki, G. , Mccoll, K. , Swann, J. R. , Kroll, J. S. , Clarke, P. , & Hall, L. J. (2020). Microbiota supplementation with *Bifidobacterium* and *Lactobacillus* modifies the preterm infant gut microbiota and metabolome: An observational study. Cell Reports Medicine, 1(5), 100077. 10.1016/j.xcrm.2020.100077 32904427 PMC7453906

[eph13146-bib-0004] Arboleya, S. , Binetti, A. , Salazar, N. , Fernández, N. , Solís, G. , Hernández‐Barranco, A. , Margolles, A. , Los Reyes‐Gavilán, C. G. , & Gueimonde, M. (2012). Establishment and development of intestinal microbiota in preterm neonates. FEMS Microbiology Ecology, 79(3), 763–772. 10.1111/j.1574-6941.2011.01261.x 22126419

[eph13146-bib-0005] Brunt, V. E. , Gioscia‐Ryan, R. A. , Casso, A. G. , Vandongen, N. S. , Ziemba, B. P. , Sapinsley, Z. J. , Richey, J. J. , Zigler, M. C. , Neilson, A. P. , Davy, K. P. , & Seals, D. R. (2020). Trimethylamine‐*N*‐oxide promotes age‐related vascular oxidative stress and endothelial dysfunction in mice and healthy humans. Hypertension, 76(1), 101–112. 10.1161/HYPERTENSIONAHA.120.14759 32520619 PMC7295014

[eph13146-bib-0006] Casadevall, A. , & Pirofski, L.‐A. (1999). Host‐pathogen interactions: Redefining the basic concepts of virulence and pathogenicity. Infection and Immunity, 67(8), 3703–3713. 10.1128/IAI.67.8.3703-3713.1999 10417127 PMC96643

[eph13146-bib-0007] Chow, J. , Tang, H. , & Mazmanian, S. K. (2011). Pathobionts of the gastrointestinal microbiota and inflammatory disease. Current Opinion in Immunology, 23(4), 473–480. 10.1016/j.coi.2011.07.010 21856139 PMC3426444

[eph13146-bib-0008] Day‐Walsh, P. , Shehata, E. , Saha, S. , Savva, G. M. , Nemeckova, B. , Speranza, J. , Kellingray, L. , Narbad, A. , & Kroon, P. A. (2021). The use of an in‐vitro batch fermentation (human colon) model for investigating mechanisms of TMA production from choline, l‐carnitine and related precursors by the human gut microbiota. European Journal of Nutrition, 60(7), 3987–3999. 10.1007/s00394-021-02572-6 33934200 PMC8437865

[eph13146-bib-0009] Eloe‐Fadrosh, E. A. , & Rasko, D. A. (2013). The human microbiome: From symbiosis to pathogenesis. Annual Review of Medicine, 64, 145–163. 10.1146/annurev-med-010312-133513 PMC3731629 23327521

[eph13146-bib-0010] Farshim, P. , Walton, G. , Chakrabarti, B. , Givens, I. , Saddy, D. , Kitchen, I. , R Swann, J. , & Bailey, A. (2016). Maternal weaning modulates emotional behavior and regulates the gut‐brain axis. Science Reports, 6, 21958. 10.1038/srep21958 PMC4763306 26903212

[eph13146-bib-0011] Frost, G. , Sleeth, M. L. , Sahuri‐Arisoylu, M. , Lizarbe, B. , Cerdan, S. , Brody, L. , Anastasovska, J. , Ghourab, S. , Hankir, M. , Zhang, S. , Carling, D. , Swann, J. R. , Gibson, G. , Viardot, A. , Morrison, D. , Louise Thomas, E. , & Bell, J. D. (2014). The short‐chain fatty acid acetate reduces appetite via a central homeostatic mechanism. Nature Communications, 5, 3611. 10.1038/ncomms4611 PMC4015327 24781306

[eph13146-bib-0012] Geng, J. , Yang, C. , Wang, B. , Zhang, X. , Hu, T. , Gu, Y. , & Li, J. (2018). Trimethylamine *N*‐oxide promotes atherosclerosis via CD36‐dependent MAPK/JNK pathway. Biomedicine & Pharmacotherapy, 97, 941–947. 10.1016/j.biopha.2017.11.016 29136772

[eph13146-bib-0013] Hales, C. N. , & Barker, D. J. P. (2001). The thrifty phenotype hypothesis. British Medical Bulletin, 60, 5–20. 10.1093/bmb/60.1.5 11809615

[eph13146-bib-0014] Hanson, M. A. , & Gluckman, P. D. (2014). Early developmental conditioning of later health and disease: Physiology or pathophysiology? Physiological Reviews, 94(4), 1027–1076. 10.1152/physrev.00029.2013 25287859 PMC4187033

[eph13146-bib-0015] Hornef, M. (2015). Pathogens, commensal symbionts, and pathobionts: Discovery and functional effects on the host. ILAR Journal, 56(2), 159–162. 10.1093/ilar/ilv007 26323625

[eph13146-bib-0016] Hsu, B. B. , Gibson, T. E. , Yeliseyev, V. , Liu, Q. , Lyon, L. , Bry, L. , Silver, P. A. , & Gerber, G. K. (2019). Dynamic modulation of the gut microbiota and metabolome by bacteriophages in a mouse model. Cell Host & Microbe, 25(6), 803–814.e805. 10.1016/j.chom.2019.05.001 31175044 PMC6579560

[eph13146-bib-0017] Johnson, C. L. , & Versalovic, J. (2012). The human microbiome and its potential importance to pediatrics. Pediatrics, 129(5), 950–960. 10.1542/peds.2011-2736 22473366 PMC3340594

[eph13146-bib-0018] Koeth, R. A. , Wang, Z. , Levison, B. S. , Buffa, J. A. , Org, E. , Sheehy, B. T. , Britt, E. B. , Fu, X. , Wu, Y. , Li, L. , Smith, J. D. , Didonato, J. A. , Chen, J. , Li, H. , Wu, G. D. , Lewis, J. D. , Warrier, M. , Brown, J. M. , Krauss, R. M. , … Hazen, S. L. (2013). Intestinal microbiota metabolism of L‐carnitine, a nutrient in red meat, promotes atherosclerosis. Nature Medicine, 19(5), 576–585. 10.1038/nm.3145 PMC3650111 23563705

[eph13146-bib-0019] Lau, S. K. P. , Woo, P. C. Y. , Woo, G. K. S. , Fung, A. M. Y. , Wong, M. K. M. , Chan, K.‐M. , Tam, D. M. W. , & Yuen, K.‐Y. (2004). *Eggerthella hongkongensis* sp. nov. and *Eggerthella sinensis* sp. nov., two novel *Eggerthella* species, account for half of the cases of *Eggerthella bacteremia* . Diagnostic Microbiology and Infectious Disease, 49(4), 255–263. 10.1016/j.diagmicrobio.2004.04.012 15313530

[eph13146-bib-0020] Lee, J. , Venna, V. R. , Durgan, D. J. , Shi, H. , Hudobenko, J. , Putluri, N. , Petrosino, J. , Mccullough, L. D. , & Bryan, R. M. (2020). Young versus aged microbiota transplants to germ‐free mice: Increased short‐chain fatty acids and improved cognitive performance. Gut Microbes, 12(1), 1–14. 10.1080/19490976.2020.1814107 PMC7757789 32897773

[eph13146-bib-0021] Li, D. , Ke, Y. , Zhan, R. , Liu, C. , Zhao, M. , Zeng, A. , Shi, X. , Ji, L. , Cheng, S.i , Pan, B. , Zheng, L. , & Hong, H. (2018). Trimethylamine‐N‐oxide promotes brain aging and cognitive impairment in mice. Aging Cell, 17(4), e12768. 10.1111/acel.12768 29749694 PMC6052480

[eph13146-bib-0022] Mars, R. A. T. , Yang, Y.i , Ward, T. , Houtti, M.o , Priya, S. , Lekatz, H. R. , Tang, X. , Sun, Z. , Kalari, K. R. , Korem, T. , Bhattarai, Y. , Zheng, T. , Bar, N. , Frost, G. , Johnson, A. J. , Van Treuren, W. , Han, S. , Ordog, T. , Grover, M. , … Kashyap, P. C. (2020). Longitudinal multi‐omics reveals subset‐specific mechanisms underlying irritable bowel syndrome. Cell, 182(6), 1460–1473.e17. 10.1016/j.cell.2020.08.007 32916129 PMC8109273

[eph13146-bib-0023] Martin, A. M. , Sun, E. W. , Rogers, G. B. , & Keating, D. J. (2019). The influence of the gut microbiome on host metabolism through the regulation of gut hormone release. Frontiers in Physiology, 10, 428. 10.3389/fphys.2019.00428 31057420 PMC6477058

[eph13146-bib-0024] Modasia, A. , Parker, A. , Jones, E. , Stentz, R. , Brion, A. , Goldson, A. , Defernez, M. , Wileman, T. , Ashley Blackshaw, L. , & Carding, S. R. (2020). Regulation of enteroendocrine cell networks by the major human gut symbiont *Bacteroides thetaiotaomicron* . Frontiers in Microbiology, 11, 575595. 10.3389/fmicb.2020.575595 33240233 PMC7677362

[eph13146-bib-0025] Mohajeri, M. H. , Brummer, R. J. M. , Rastall, R. A. , Weersma, R. K. , Harmsen, H. J. M. , Faas, M. , & Eggersdorfer, M. (2018). The role of the microbiome for human health: From basic science to clinical applications. European Journal of Nutrition, 57(1), 1–14. 10.1007/s00394-018-1703-4 PMC5962619 29748817

[eph13146-bib-0026] Montassier, E. , Valdés‐Mas, R. , Batard, E. , Zmora, N. , Dori‐Bachash, M. , Suez, J. , & Elinav, E. (2021). Probiotics impact the antibiotic resistance gene reservoir along the human GI tract in a person‐specific and antibiotic‐dependent manner. Nature Microbiology, 6(8), 1043–1054. 10.1038/s41564-021-00920-0 PMC8318886 34226711

[eph13146-bib-0027] Osman, A. , Zuffa, S. , Walton, G. , Fagbodun, E. , Zanos, P. , Georgiou, P. , Kitchen, I. , Swann, J. , & Bailey, A. (2021). Post‐weaning A1/A2 β‐casein milk intake modulates depressive‐like behavior, brain μ‐opioid receptors, and the metabolome of rats. iScience, 24(9), 103048. 10.1016/j.isci.2021.103048 34585111 PMC8450247

[eph13146-bib-0028] Parker, A. , Romano, S. , Ansorge, R. , Aboelnoer, A. , Le Gall, G. , Savva, G. M. , Telatin, A. , Jones, E. , Baker, D. , Rudder, S. , Blackshaw, L. A. , Jeffery, G. & Carding, S. R. (2021). Heterochronic fecal microbiota transfer reverses hallmarks of the aging murine gut, eye and brain. SSRN Electronic Journal. 10.2139/ssrn.3811833 PMC9063061 35501923

[eph13146-bib-0029] Pérez‐Reytor, D. , Puebla, C. , Karahanian, E. , & García, K. (2021). Use of short‐chain fatty acids for the recovery of the intestinal epithelial barrier affected by bacterial toxins. Frontiers in Physiology, 12(721), 650313. 10.3389/fphys.2021.650313 34108884 PMC8181404

[eph13146-bib-0030] Rajakovich, L. J. , Fu, B. , Bollenbach, M. , & Balskus, E. P. (2021). Elucidation of an anaerobic pathway for metabolism of l‐carnitine‐derived gamma‐butyrobetaine to trimethylamine in human gut bacteria. Proceedings of the National Academy of Sciences, USA, 118(32), e2101498118. 10.1073/pnas.2101498118 PMC8364193 34362844

[eph13146-bib-0031] Rios‐Covian, D. , González, S. , Nogacka, A. M. , Arboleya, S. , Salazar, N. , Gueimonde, M. , & De Los Reyes‐Gavilán, C. G. (2020). An overview on fecal branched short‐chain fatty acids along human life and as related with body mass index: Associated dietary and anthropometric factors. Frontiers in Microbiology, 11, 973. 10.3389/fmicb.2020.00973 32547507 PMC7271748

[eph13146-bib-0032] Senthong, V. , Wang, Z. , Fan, Y. , Wu, Y. , Hazen, S. L. , & Tang, W. H. W. (2016). Trimethylamine *N*‐oxide and mortality risk in patients with peripheral artery disease. Journal of the American Heart Association, 5(10), e004237. 10.1161/JAHA.116.004237 27792653 PMC5121520

[eph13146-bib-0033] Stearns‐Kurosawa, D. J. , Osuchowski, M. F. , Valentine, C. , Kurosawa, S. , & Remick, D. G. (2011). The pathogenesis of sepsis. Annual Review of Pathology, 6, 19–48. https://www.annualreviews.org/doi/10.1146/annurev‐pathol‐011110‐130327 10.1146/annurev-pathol-011110-130327 PMC3684427 20887193

[eph13146-bib-0034] Suez, J. , Zmora, N. , Zilberman‐Schapira, G. , Mor, U. , Dori‐Bachash, M. , Bashiardes, S. , Zur, M. , Regev‐Lehavi, D. , Ben‐Zeev Brik, R. , Federici, S. , Horn, M. , Cohen, Y. , Moor, A. E. , Zeevi, D. , Korem, T. , Kotler, E. , Harmelin, A. , Itzkovitz, S. , Maharshak, N. , … Elinav, E. (2018). Post‐antibiotic gut mucosal microbiome reconstitution is impaired by probiotics and improved by autologous FMT. Cell, 174(6), 1406–1423.e16. 10.1016/j.cell.2018.08.047 30193113

[eph13146-bib-0035] Suzuki, T. , Heaney, L. M. , Bhandari, S. S. , Jones, D. J. L. , & Ng, L. L. (2016). Trimethylamine *N*‐oxide and prognosis in acute heart failure. Heart, 102(11), 841–848. 10.1136/heartjnl-2015-308826 26869641

[eph13146-bib-0036] Suzuki, T. , Yazaki, Y. , Voors, A. A. , Jones, D. J. L. , Chan, D. C. S. , Anker, S. D. , Cleland, J. G. , Dickstein, K. , Filippatos, G. , Hillege, H. L. , Lang, C. C. , Ponikowski, P. , Samani, N. J. , Van Veldhuisen, D. J. , Zannad, F. , Zwinderman, A. H. , Metra, M. , & Ng, L. L. (2019). Association with outcomes and response to treatment of trimethylamine N‐oxide in heart failure: Results from BIOSTAT‐CHF. European Journal of Heart Failure, 21(7), 877–886. 10.1002/ejhf.1338 30370976

[eph13146-bib-0037] Swann, J. R. , Spitzer, S. O. , & Diaz Heijtz, R. (2020). Developmental signatures of microbiota‐derived metabolites in the mouse brain. Metabolites, 10(5), 172. 10.3390/metabo10050172 PMC7281085 32344839

[eph13146-bib-0038] Tan, Y. , Sheng, Z. , Zhou, P. , Liu, C. , Zhao, H. , Song, L. , Li, J. , Zhou, J. , Chen, Y. , Wang, L. , Qian, H. , Sun, Z. , Qiao, S. , Xu, B. , Gao, R. , & Yan, H. (2019). Plasma trimethylamine N‐oxide as a novel biomarker for plaque rupture in patients with ST‐segment‐elevation myocardial infarction. Circulation Cardiovascular Interventions, 12(1), e007281. 10.1161/circinterventions.118.007281 30599768

[eph13146-bib-0039] Vieira‐Silva, S. , Falony, G. , Darzi, Y. , Lima‐Mendez, G. , Garcia Yunta, R. , Okuda, S. , Vandeputte, D. , Valles‐Colomer, M. , Hildebrand, F. , Chaffron, S. , & Raes, J. (2016). Species‐function relationships shape ecological properties of the human gut microbiome. Nature Microbiology, 1(8), 16088. 10.1038/nmicrobiol.2016.88 27573110

[eph13146-bib-0040] Vuong, H. E. , Pronovost, G. N. , Williams, D. W. , Coley, E. J. L. , Siegler, E. L. , Qiu, A. , Kazantsev, M. , Wilson, C. J. , Rendon, T. , & Hsiao, E. Y. (2020). The maternal microbiome modulates fetal neurodevelopment in mice. Nature, 586(7828), 281–286. 10.1038/s41586-020-2745-3 32968276 PMC7554197

[eph13146-bib-0041] Wang P.‐X. , Deng X.‐R. , Zhang C.‐H. , & Yuan H.‐J. (2020). Gut microbiota and metabolic syndrome. Chinese Medical Journal, 133(7), 808–816. 10.1097/cm9.0000000000000696 32106124 PMC7147654

[eph13146-bib-0042] Zhang, S. , Dogan, B. , Guo, C. , Herlekar, D. , Stewart, K. , Scherl, E. J. , & Simpson, K. W. (2020). Short chain fatty acids modulate the growth and virulence of pathosymbiont escherichia coli and host response. Antibiotics, 9(8), 462. 10.3390/antibiotics9080462 PMC7460008 32751519

[eph13146-bib-0043] Zhang, Z. , Tang, H. , Chen, P. , Xie, H. , & Tao, Y. (2019). Demystifying the manipulation of host immunity, metabolism, and extraintestinal tumors by the gut microbiome. Signal Transduction and Targeted Therapy, 4(1), 41. 10.1038/s41392-019-0074-5 31637019 PMC6799818

[eph13146-bib-0044] Zmora, N. , Zilberman‐Schapira, G. , Suez, J. , Mor, U. , Dori‐Bachash, M. , Bashiardes, S. , Kotler, E. , Zur, M. , Regev‐Lehavi, D. , Brik, R. B.‐Z. , Federici, S. , Cohen, Y. , Linevsky, R. , Rothschild, D. , Moor, A. E. , Ben‐Moshe, S. , Harmelin, A. , Itzkovitz, S. , Maharshak, N. , … Elinav, E. (2018). Personalized gut mucosal colonization resistance to empiric probiotics is associated with unique host and microbiome features. Cell, 174(6), 1388–1405.e21. 10.1016/j.cell.2018.08.041 30193112

